# *Chlamydia psittaci* in Faecal Samples of Feral Pigeons (*Columba livia* forma *urbana*) in Urban Areas of Lublin city, Poland

**DOI:** 10.1007/s00284-022-03072-4

**Published:** 2022-10-17

**Authors:** Katarzyna Kowalczyk, Angelina Wójcik-Fatla

**Affiliations:** Department of Health Biohazards and Parasitology, Institute of Rural Health, Jaczewskiego 2, 20-090 Lublin, Poland

## Abstract

Pigeons are a typical host and natural reservoir of *Chlamydia psittaci*, the etiological agent of avian chlamydiosis, considered as a neglected zoonotic diseases. The aim of the study was to determine the prevalence of *C. psittaci* in faecal samples of feral pigeons (*Columba livia* forma *urbana*) as a potential source of infection related to the presence of synanthropic birds in urban areas. A total of 143 samples of dry and fresh faeces of feral pigeons, were collected in the city of Lublin (Poland), from April to September 2021. Molecular detection of *C. psittaci* was performed by nested-PCR and real-time PCR, confirmed by sequencing. Among the collected samples, 5 positive results were obtained in nested-PCR (3.5%), while in real-time PCR, the number of positive samples increased to 11 (7.7%). The positive samples showed 100% identity to the *C. psittaci* strain AMK (CP047319.1). *C. psittaci* was found in 7 out of 111 (6.3%) faecal samples collected in public places, and in 4 out of 32 (12.5%) samples from the nesting site (4.9% and 2.8% among a total of 143 samples, respectively). The infection was detected in both dry and fresh faeces (9.1% and 4.5%, respectively). The highest number of positive results was obtained in June—5 (3.5%). Feral pigeons occurring in urban areas are a natural reservoir of *C. psittaci* posing a potential risk of zoonotic infections. However, further studies on exposure to contaminated pigeon faeces in terms of occupational and non-occupational risk of chlamydiosis are needed.

**Supplementary Information:**

The online version contains supplementary material available at 10.1007/s00284-022-03072-4.

## Introduction

*Chlamydia psittaci* belongs to Gram-negative obligate intracellular bacteria of the *Chlamydiaceae* family with 13 other recognised species, such as *C*. *pneumoniae*, *C*. *abortus*, *C*. *felis*, *C*. *suis* [1], classified to the *Chlamydia* genus based on 16S rRNA gene sequence analysis, high genomic similarity, and distinctive phenotypic features [2]. Both wild and domestic birds are the typical host and natural reservoir of *C. psittaci* [3], and are the main source of human infections, mainly associated with infected parrots and pigeons [4]. Currently, 15 genotypes of *C. psittaci* have been identified in the avian reservoir and considered potentially pathogenic for human health [5].

Pathogen transmission occurs as a result of close contact with infected birds or inhaling bioareosol containing their secretions: faecal dust, feather particles or dried secretions from the eyes and the respiratory tract [6, 7]. Human ornithosis, also called psittacosis or parrot fever, is mainly reported as subclinical or brief, self-limiting, influenza-like illness, whereas cases of fulminant psittacosis with multi-organ failure are noted rarely [8]. In Europe, up to 400 cases of chlamydiosis are reported annually, while in the USA the number of cases does not exceed 11, on average [6, 9]. In Poland, a total of only 23 cases were reported between 2000 and 2019 [10].

From time to time, epidemiological small outbreaks of ornithosis are noted, mainly related to recreational or occupational exposure (e.g., among workers at chicken slaughter plants, pet shop or zoo employees, veterinary surgeons, and bird breeders) [8, 9].

One of the main sources of *C. psittaci* infection in towns, in terms of both occupational and no-occupational risk, are free-living and synanthropic pigeons existing in urban areas [11].

Studies on the prevalence of *C. psittaci* in feral pigeons in some European towns and cities revealing a seropositivity ranged from 19.4 to 95.6%, while bacteria DNA was detected from 3.4 to 50% of the tissue samples, cloacal swabs, and faecal droppings [11].

Taking into consideration that avian chlamydiosis belongs to the one of neglected zoonotic diseases (NZD) [12], the aim of the study was to determine the prevalence of *C. psittaci* in faecal samples of feral pigeons (*Columba livia* forma *urbana*) as a potential source of infection related to the presence of synanthropic birds in urban areas.

## Materials and Methods

### Samples Collection

A total of 143 samples of dry and fresh faeces (44 and 99, respectively) from feral pigeons (*Columba livi*a forma *urbana*), were collected in the city of Lublin, Poland, in April to September 2021. The sampling was preceded by observations of feral pigeons and selections of places characterised by the occurrence of groups of at least 10 individuals. All locations were situated in public places accessible to both birds and people (near bus stops, shops, playgrounds, outdoor gyms, schools, universities, hospitals, cemeteries), in residential areas, parks, city squares and in the city centre areas (Table 1). Observation was carried out of one selected site, a public building inhabited by a pair of pigeons and their offspring. A total of 32 faecal samples were collected from this particular site. The pigeons nested 4 times, having from 1 to 3 chicks per brood in the nest. The samples were collected during the absence of the adults in order not to interfere with the ongoing breeding season. All faecal samples were collected with a wooden disposable ENT (Ears, Nose, and Throat) spatula and placed individually into sterile tubes. The samples were immediately delivered to the laboratory for further investigations.

**Table 1:** Prevalence of *Chlamydia psittaci* in faecal samples of feral pigeons (*Columba livia* forma *urbana*) in Lublin region

Collection sites	*Chlamydia pstittaci* in faecal samples of feral pigeons [No.*/ total (%)]		
	Type of sample		Total
	fresh faeces	dry faeces	
nesting site	0/3 (0.0)	4/29 (13.8)	4/32 (12.5)
residential area	0/11 (0.0)	1/16 (6.3)	1/27 (3.7)
city square	1/14 (7.1)	1/9 (11.1)	2/23 (8.7)
park	1/3 (33.3)	1/3 (33.3)	2/6 (33.3)
city centre	0/1 (0.0)	0/4 (0.0)	0/5 (0.0)
other	0/12 (0.0)	2/38 (5.3)	2/50 (4.0)
Total	2/44 (4.5)	9/99 (9.1)	11/143 (7.7)

^*^‘No.’ number of positive samples in real-time PCR

### DNA Extraction

Total DNA from dry and fresh faeces samples each probe of 100 mg was isolated using the commercial Syngen Stool Mini Kit (Syngen Biotech, Poland), according to the manufacturer's instructions. The DNA concentration (ng/µl) and DNA purity (value 260/280) were measured using a QIAxpert spectrophotometer (Qiagen, USA). The amount of isolated DNA from all samples ranged from 0.10 to 314.32 ng/µl. For real-time PCR positive samples, the amount of isolated DNA ranged from 2.19 to 44.71 ng/µl. The purity (A_260_/A_280_) of the samples ranged from 1.14 to 3.85. DNA extracts were stored at − 20 °C until further molecular research.

### Molecular Detection of *Chlamydia psittaci* by Nested PCR

Detection of *C. psittaci* was carried out using the nested-PCR method performed according to the procedure, and cycling conditions described previously by Messmer et al. [13], with some modification. The volume reaction of 25 µl contained 1 U Taq DNA Polymerase and buffer with 15 mM MgCl2 (Qiagen, USA), 0.2 mM of each dNTP, 0.2 µM of each primer specific for 16S rRNA, 2,5 µl of DNA template for the first reaction, or 2 µl of the amplification product in the second reaction, and nuclease-free water. The positive control was DNA of *C. psittaci* isolated from Chlamydia MIF IgG kit slides (Focus Technologies, Cypress, (CA), USA) containing antigen spots consisting of elementary bodies (EBs) suspended in a yolk sac matrix, the negative control was nuclease-free water. The PCR reactions were performed using C1000 Thermal Cycler (BioRad, USA) and Mastercycler®nexus (Eppendorf, Germany). Amplification products for each step of PCR were analysed by electrophoresis on 2% agarose gel (Basica LE, Prona, Spain). The products of 436 bp and 127 bp were considered positive for the *Chlamydia* genus and *C. psittaci*, respectively.

### Molecular Detection of *Chlamydia psittaci* by Real-Time PCR

The real-time PCR reactions were performed using the *Chlamydophila psittaci* (*C. psittaci*) Real-time PCR Kit, RUO (Nzytech, Portugal), in accordance with the manufacturer’s protocols designed for the in vitro quantitative of *C. psittaci* genomes. DNA was detected by amplification of CPSIT_0607 fragment gene with the primers adapted by the manufacturer from the article by Opota et al. [14] (supplementary Table 1). Each reaction (20 µl) consisted of 10 µl 2 × Lyo NZYSupreme qPCR master mix, 1 µl *C. psittaci* primer/probe mix specific for CPSIT_0607, 1 µl Internal extraction control primer/probe mix, 3 µl RNase/DNase free water and 5 µl of DNA template. The amplification was carried out in Step One Real-Time PCR System (Applied Biosystems, Inc., Foster City (CA), USA) under the following conditions: polymerase activation at 95 °C for 2 min and 50 cycles, each of 5 s at 95 °C and 30 s at 60 °C. The standard curve was prepared from 6 points: positive control (2 × 10^5^) and its subsequent tenfold dilutions (2 × 10^4^, 2 × 10^3^, 2 × 10^2^, 20, 2). In the presence/absence experiment, 12 out of 143 samples were confirmed as positive. However, in the quantitation – standard curve experiment, in one of the samples the amount of DNA was undetermined. Therefore, 11 samples of pigeon faeces were considered positive (11/143, 7.7%).

### DNA Sequencing

Sequencing of PCR products were performed with the ABI PRISM 310 Genetic Analyzer (Applied Biosystems, USA) using a BigDye® Terminator v3.1 Cycle Sequencing Kit and a Big Dye XTerminator Purification Kit (Applied Biosystems, USA). The nucleotide sequences were compared with data stored in GenBank using the Basic Local Alignment Search Tool (BLAST).

## Results and Discussion

Over the course of many decades, wild birds, including feral pigeons, have adapted to life in a specific and dynamically changing urban environment, and use optimal conditions for reproduction and growth almost all year round. The developing green infrastructure of cities (plantings, trees, construction of water reservoirs, etc.) will contribute to the increasing of ecosystem biodiversity, which undoubtedly has a beneficial effect on humans. Nevertheless, one should be aware of the existence of various undesirable health hazards, such as pathogens transmitted by rats, ticks or pigeons [15]. The density and maintenance of the pigeon population in an urban agglomeration is closely related to human activity in terms of urban spatial development, as well as human behaviours, e.g., intentional ‘mercy feeding’ [16].

In Poland, large populations of feral pigeon (*Columba livia* forma *urbana*) are commonly distributed within the urban spaces, in many public areas such as parks, city squares, promenades, residential areas, bus stops and railway stations, which provide access to food, nesting sites and protection against predators [17]. However, the presence of feral pigeons is associated with the risk of harmful biological agents, including at least 60 microorganisms potentially pathogenic for humans [18]. Among them, *Cryptosporiudium* spp. [19], *Campylobacter jejuni* [20], *Escherichia coli* [21] and zoonotic yeast [22] have been detected. The pigeons are also the second important reservoir of *C. psittaci*, the agent of human psittacosis [4, 23], while in general psittacine birds remain the most important reservoir of the pathogen [3, 4].

So far, there is little research on feral pigeon contamination with *C. psittaci* in Poland, especially among the urban population, as a potential threat to public health. In this study, the highest percentages were obtained in samples from areas with the observed highest density of feral pigeons and high availability of shelters and nesting places, including parks, a square located near a hospital, and residential areas overgrown with numerous trees (Table 1). The average prevalence of *C. psittaci* in the examined individual faecal samples of feral pigeons was on the medium level (overall 11/143, 7.7% in real-time PCR), compared to the results ranging from 2.4 to 14.3% obtained in some other cities (Table 4), but higher than the European average of 5.7% [24]. Moreover, the prevalence of *C. psittaci* in pigeons from other regions in Poland was also lower compared to this study. Specifically, Stenzel et al. [25] found 3.9% of infected individuals among the feral population, while Szymańska-Czerwińska et al. [5] detected *Chlamydiaceae* in 4.7% samples of cloacal or faecal swabs in the Columbidae family, but *C. psittaci* DNA was confirmed only in 1 out of 64 tested pigeons. These lower percentages are closer to our results obtained in nested-PCR, where only 5 samples were positive (5/143, 3.5%). The sequences of amplification products (127 bp) were compared with the sequences deposited in the GenBank. All 5 positive samples showed 100% identity to the *C. psittaci* strain AMK (GenBank Accession No. CP047319.1) [26]. However, the obtained sequences could not be placed in GenBank because of too short amplicons (more than 200 nucleotides required), whereas the products of the first amplification (436 bp) were not visible on agarose gel (Table 2).

**Table 2:** Summary of the positive faecal samples for *Chlamydia psittaci*

No. of the sample	Place of origin	Month of collection	Results of PCR		Result of real-time PCR
			PCR (436 bp)	nested-PCR (127 bp)	copy number [per µl]
No. 1	nesting site	April	−	+	11.35
No. 2	other	April	−	+	0.15
No. 3	nesting site	April	−	+	0.7
No. 4	city square	June	−	−	1.99
No. 5	park	June	−	−	2.55
No. 6	park	June	−	−	16.3
No. 7	other	June	−	−	3.27
No. 8	residential area	June	−	−	1.8
No. 9	city square	August	−	−	18.99
No. 10	nesting site	September	−	+	0.65
No. 11	nesting site	September	−	+	3.71

Research conducted in Poland showed that the higher results were obtained among ornamental or domestic pigeons (approx. 10%), which were much more exposed to stress factors (e.g., competition flights, exhibitions, transport), which could induce faecal or nasal excretion of *Chlamydia* [25].

The shedding of *C. psittaci* can be activated and/or enhanced by the occurrence of other stressors, such as the breeding season, bird infections or infestations [24, 25, 27]. One of the most common pathogens affecting the pigeon population is circovirus (PiCV), considered to be especially dangerous for young specimens (YPDS – Young Pigeon Disease Syndrome) [28]. Moreover, intermittent shedding of *C. psittaci* with faeces or body secretions may cause false negative results [24, 29].

The variability between prevalences in different studies could be due to several causes, including of sample type (e.g., cloacal or pharyngeal swabs, faeces, organ, tissue), different procedures and laboratory methods (serological, molecular, cell culture), sampling period, distribution and density of birds in urban settings [11, 27, 30]. Referring to data from several other cities or towns, the prevalence of *C. psittaci* in feral pigeon populations were in the range from 0.7 to 63.3% (Table 4).

Heddema et al. [31] showed a dependence of pathogen shedding intensity associated with the breeding season, during which *C. psittaci* excretion was twice as high (10%) compared to the low-breeding season (5.0%). In the current study, *C. psittaci* was found in 7 out of 111 (6.3%) faecal samples collected in urban areas and in 4 out of 32 (12.5%) samples from a nesting site, but the too small a number of samples in each group did not allow for clear confirmation of such a correlation. Moreover, it is not known whether samples taken outside the nesting site were not obtained from pigeons during the breeding season. Besides, the capacity of pigeons to breed throughout the year depends on various factors, such as availability of food and shelter in cities, as well as behavioural changes [11].

In this study, almost all faecal samples were collected in spring and summer—the period of the highest breeding activity of pigeons in Poland (Table 3). The highest number of positive results was obtained in June – 5/143 (3.5%). Scientific research has confirmed that *C. psittaci* can survive in a relatively wide temperature range. Wannaratana et al. [32] demonstrated that *C. psittaci* can survive at 56 °C for up to 72 h with preservation of infectivity. Kramer et al. [33] reported that bacteria could survive on dry surfaces for up to 15 days at 4 °C, which may suggest that the possibility of infection exists in mild winters. Pigeon faeces are also the matrix that covers and protects microorganisms, and consequently they can survive longer despite exposure to a number of changing and unfavourable environmental factors [24, 32].

**Table 3:** Prevalence of *Chlamydia psittaci* in faecal samples of feral pigeons (*Columba livia* forma *urbana*) in collection months

Place of origin	Collection month [No.*/ total (%)]					
	April	June	July	August	September	Total
nesting site	2/4 (50.0)	0/2 (0.0)	0/2 (0.0)	0/2 (0.0)	2/22 (9.1)	4/32 (12.5)
residential area	0/3 (0.0)	1/12 (8.3)	0/0 (0.0)	0/12 (0.0)	0/0 (0.0)	1/27 (3.7)
city square	0/0 (0.0)	1/8 (12.5)	0/0 (0.0)	1/15 (6.7)	0/0 (0.0)	2/23 (8.7)
park	0/0 (0.0)	2/4 (50.0)	0/0 (0.0)	0/2 (0.0)	0/0 (0.0)	2/6 (33.3)
city centre	0/0 (0.0)	0/0 (0.0)	0/0 (0.0)	0/5 (0.0)	0/0 (0.0)	0/5 (0.0)
other	1/21 (4.8)	1/13 (7.7)	0/4 (0.0)	0/2 (0.0)	0/10 (0.0)	2/50 (4.0)
Total	3/28 (10.7)	5/39 (12.8)	0/6 (0.0)	1/38 (2.6)	2/32 (6.3)	11/143 (7.7)

^*^ ‘No.’ number of positive samples in real-time PCR

In the current study, *C. psittaci* DNA was found twice a year (April and September) in the selected nesting site (Table 2). *Chlamydia* appears to be more likely to persist in pigeon faeces mainly deposited at the breeding, roosting and feeding sites that are usually sheltered from adverse weather conditions. This type of pigeon nests within residential and public buildings could be a potential source of human psittacosis related to occupational (e.g., cleaners, employees of the city cleaning department, demolition/construction workers) and non-occupational exposure (e.g., residents, feeding and care of feral pigeon) in urban areas [7, 11].

In the case of city pigeons, it is much more difficult to determine a direct relationship between exposure to contaminated faeces and the acquisition of infection compared to e.g., poultry farmers, who have contact with an avian source [34]. In urban spaces, exposure of zoo employees and veterinarians has been determined [35, 36], but among other groups having occasional or accidental contact with pigeon faeces, reports of *C. psittaci* infections are rare. Mair-Jenkins et al. [37] described a small psittacosis outbreak among office workers for which the probable source of zoonotic transmission was indirect environmental contact with *C. psittaci* from roosting sites of feral pigeons. In contrast, Sachse et al. [24] showed that none of the community workers were infected with *C. psittaci* despite daily contact with feral pigeons. On the other hand, the removal of pigeon faeces is usually done with the use of water or detergents, while the main route of infection is the inhalation of dry bird excreta and secretions containing *C. psittaci* [38].

The results obtained in the current study show that despite the difference between the percentage of positive results in dry (9/99, 9.1%) and fresh (2/44, 4.5%) faecal samples, the results proved to be statistically insignificant (Fisher’s exact test; *p* < 0.05) (Table 1). Indirect environmental exposure is presumed to be an important factor of human infections [8, 39], even if contact with pigeon faeces is temporary [38]. In addition, cases caused by direct contact with sick or dead pigeons cannot be excluded [18], but the most important seems to be the tendency of pigeon faeces to dust formation and inhalation. Studies on the detection of *C. psittaci* in air samples taken from the vicinity of nests located close to windows or air conditioning systems in buildings, could assist in the risk of human chlamydiosis by the inhalation route. It would also be worthwhile to conduct research on the survival of *Chlamydia* spp. in the environment, including the distinction between viable and non-viable forms. Little research has been conducted to date on the persistence and infectivity of *C. psittaci* under natural conditions [32, 33].

Accumulation of pigeon faeces in public areas contributes to the spread of zoonotic agents in the environment, and that this host may contribute to the shedding of pathogens in many places (parks, city squares, benches, pavements, buildings, monuments) [7, 11] Most of the positive results in the current study concerned overcrowded places with a high concentration of pigeon populations. Geigenfeind et al. [7] suggested that proper management of feral pigeon populations in cities through abundance reduction could have a beneficial effect on the health status of the birds and a lower rate of *C. psittaci* infections in feral pigeons. Some authors have indicated the positive aspects of using the feral pigeon population as bioindicators of heavy metal pollutions [40], and for monitoring zoonotic pathogens in urban areas [41]. Despite rare cases of human psittacosis and the low prevalence of *C. psittaci* among pigeons determined in some cities (Table 4), the obtained level of faecal contamination in the current study confirms a potential risk of chlamydiosis in urban areas.

**Table 4:** Prevalence of *Chlamydia psittaci* among the feral pigeon population (*Columba livia* forma *urbana*) in urban areas (derived from the literature)

Country	Town or city	Year of sampling	Period of sampling	Type of samples	Molecular methods (target gen)	Results positive/ total (% positive)	Reference
Iran	unknown	–	–	fresh faecal	PCR (*ompA*)	64/445 (14.3%)	[42]
	Ahvaz	–	hot and cold seasons	c-swab	PCR (*pmp*)	2/280 (0.7%)	[43]
Slovakia	unknown	–	summer period	c-swab, p-swab	PCR (23S rRNA)	13/122 (10.7%)^a^	[23]
Switzerland	Basel	2007—2008	February (low-breeding period)	c-swab, p-swab	nested PCR (*ompA*)	17/202 (8.4%)^b^	[7]
		2008—2009	July – May (breeding period)				
		2008—2009	all year	fresh faecal		0/520 (0.0%)	
	Lucerne	–	-	c-swab	ArrayTube microarray assay (23S rRNA); PCR (16S rRNA)	2/60 (3.3%)^c^	[44]
	Zurich					10/24 (41.7%)^d^	
	Berne, Lucerne, Zurich, various rural places	2014–2018	–	c-swab, c/c-swab***, liver	DNA microarray assay PCR (23S rRNA); PCR (16S rRNA); real-time PCR (*ompA*)	62/323 (19.2%)^e^	[27]
Netherlands	Utrecht	2017	May	fresh faecal	real-time PCR	1/41 (2.4%)^f^	[45]
	Haarlem					3/40 (7.5%)^f^	
	Amsterdam	2005	February – March (low-breeding period)	fresh faecal	real-time PCR (*ompA*)	8/160 (5.0%)	[31]
			May (breeding period)			18/171 (10.0%)	
			Total			26/331 (7.9%)^g^	
Belgium	Ghent	2008	winter period	p-swab	nested PCR (*ompA*)	1/61 (1.6%)	[29]
Germany	Moers	2009	April and May	c-swab	real-time PCR (*ompA*)	59/570 (10.4%)^h^	[24]
		2010	May and June	pooled faeces		38/60 (63.3%)^h^	
France	Paris, Clamart, Pantin, Courbevoie, Creil, Gennevilliers, Fontenay-sous-Bois	2009	February – May	c-swab	real-time PCR (*ompA*)	85/708 (12.0%)	[46]
Spain	Madrid	2005–2010	July—December	c-swab, p-swab	PCR; real-time PCR	4/156 (2.56%)	[30]
		2010–2014				191/251 (4.36–12.94%)^#^	

^*^‘c-swab’—cloacal swab samples; ** ‘p-swab’—pharyngeal swabs samples; *** ‘c/c-swab’ – combined choanal/cloacal swab samples; ^#^—range of prevalence in pooled samples

^a^ two isolates belonged to genotype B

^a^ all isolates were closed to genotype B and E

^b^ seven isolates belonged to genotype B and one isolates was mixed genotypes A, B, E/B

^c^ one isolate belonged to genotype B

^d^ five isolates belonged to genotype B and one isolate was genotype E

^e^ including one sample mixed with *C. psittaci* and *C. avium*

^f^ all isolates belonged to genotype B

^g^ ten isolates belonged to genotype B

^h^ in total 85 strains belonging to genotype B and three isolates were genotype

### Limitation of the Study

The study covered a relatively small number of examined feral pigeon faeces related to a wide area of research, especially that out of the total number of 143 samples, 32 (22.4%) were taken from one site. Therefore, on this basis, the degree of chlamydia infection of local populations of pigeons in the urban agglomeration of Lublin could not be determined. Moreover, the faecal samples were not collected in all months of the year, but only from April to September (excluding May), which does not confirm the exposure to *C. psittaci* throughout the year. The limitation of the used molecular methods in this study is the lack of information about the viability of the pathogen in the analysed material.

## Conclusion

The study confirmed that feral pigeons occurring in urban areas are a natural reservoir of *C. psittaci,* causing a potential risk of zoonotic infections. Further studies are needed on exposure to contaminated pigeon faeces in terms of occupational and non-occupational risk of chlamydiosis. The obtained results suggest that *C. psittaci* may persist in feral pigeons and in an urban environment for most of the year.

## Supplementary Information

Below is the link to the electronic supplementary material.

Supplementary file1 (DOCX 27 kb)

## Data Availability

All data generated or analysed during this study are included in this published article.
