# Supplementary material for: Chlamydia psittaci in Faecal Samples of Feral Pigeons (Columba livia forma urbana) in Urban Areas of Lublin city, Poland
Source: Curr Microbiol. 2022 Oct 17;79(12):367. doi: 10.1007/s00284-022-03072-4 (PMC9576673; doi:10.1007/s00284-022-03072-4)
Supplement: Supplementary file 1 — Supplementary file1 (DOCX 27 kb) [file 284_2022_3072_MOESM1_ESM.docx]

**Supplementary Table 1.** Oligonucleotides used in the study

| **Assay format** | **Target gene** | **Oligonucleotide** | **Primer sequence 5′–3′** | **Amplicon size [bp]** | **References** |
| --- | --- | --- | --- | --- | --- |
| Nested PCR (first-step) | 16S rRNA | Forward | ACGGAATAATGACTTCGG | 436 | [13] |
|  |  | Reverse | TACCTGGTACGCTCAATT |  |  |
| Nested PCR (second-step) |  | Forward | ATAATGACTTCGGTTGTTATT | 127 |  |
|  |  | Reverse | TGTTTTAGATGCCTAAACAT |  |  |
| Real-time PCR | CPSIT_0607 | CPSI_00F | AGCATTAGCCAGCGCTTTAGA | 118 | [14] |
|  |  | CPSI_00R | TCTCTGAGCAAAAACACTGCGT |  |  |
|  |  | CPSI_00R_C147G | TCTCTCAGCAAAAACACTGCGT |  |  |
|  |  | CPSI_00P_MGB | ACAAAGACCTGGCGAGTA |  |  |

**Supplementary Table 2.** Data on the design of the experiment

| Experimental design | |
| --- | --- |
| Definition of experimental and control groups | Experimental group consisted of dry and fresh feral pigeon faeces.  No control group appeared in the study. |
| Number within experimental group. | A total of 143 samples including dry or fresh pigeon faeces in amount of 44 and 99, respectively. |
| Assay carried out by the core or investigator’s laboratory? | Investigator’s laboratory: Department of Health Biohazards and Parasitology of Institute of Rural Health, Poland. |
| Sample | |
| Description | The feral pigeon faeces collected in the city of Lublin, Poland, from April to September in 2021. |
| Volume/mass of sample processed | 100 mg |
| Processing procedure | The samples were collecting with a wooden disposable ENT spatula into sterile tubes and intended for nucleic acid (DNA) isolation immediately after delivering to the laboratory, without keeping at low temperatures. |
| Nucleic acid extraction | |
| Procedure and/or instrumentation | Instrumentation:  - analytical balance of 0.0001 g accuracy (Sartorius, Germany)  - ThermoMixer C (Eppendorf, Germany)  - Vortex IR (Starlab, Germany)  - Centrifuge Sigma 4-16S (Sigma, Germany)  According to manufacturer’s protocol:  - 100 mg of faecal sample was weighed in a 1.5 ml microcentrifuge tube and placed on ice;  - 300 µl Buffer DLS_1_ and 20 µl of Proteinase K added. Continuously vortexed for 5 min;  - incubated at 60 °C for 20 min;  - centrifuged briefly to remove the drops from the lid of the tube;  - the samples were brought to room temperature (15-25 °C);  - 300 µl Buffer DLS_2_ was added, vortexed and incubated on ice for 5 min;  - centrifuged for 5 min at 18.000 × *g*;  - the supernatant was transferred to a new 1.5 ml tube. 200 µl Buffer DLS_3_ added, vortexed and incubated at room temperature (15-25 °C) for 2 min;  - centrifuged for 2 min at 18.000 × *g*;  - 250 µl the supernatant was transferred to a new 1.5 ml tube. 250 µl Buffer DWS, 250 µl ethanol added and pulsed vortexed;  - spin column DS provided in the kit was placed in a new 2.0 ml collection tube. 750 µl lysate applied to spin column DS and centrifuged for 1 min at 18.000 × *g*;  - spin column DS was placed in a new 2.0 ml collection tube, 750 µl Buffer DPZ added and centrifuged for 1 min at 18.000 × *g* (step repeated);  - centrifuged Spin column DS for 3 min at 18.000 × *g*;  - spin column DS was transferred into a new 1.5 ml microcentrifuge tube, pipetted 100 µl Buffer DE (previously heated to 60 °C) and incubated for 2 min at room temperature;  - centrifuged for 1 min at 18.000 × *g* to elute DNA. |
| Name of kit and details of any modifications | Syngen Stool Mini Kit (Syngen Biotech, Poland) cat. no.: SY271010, according to the manufacturer’s instructions without any modifications. |
| Contamination assessment (DNA) | Yes |
| Nucleic acid quantification | 0.10- 314.32 ng/µl (range for all samples)  2.19- 44.71 ng/µl (range for all real-time PCR positive samples) |
| Instrument and method | QIAxpert spectrophotometer (Qiagen, USA) |
| Purity (A_260_/A_280_) | 1.14- 3.85 |
| qPCR target information | |
| Gene symbol/ qPCR oligonucleotides | Target gene: CPSIT_0607 (*Chlamydia psittaci*; amplicon size 118 bp):  - CPSI_00F: AGCATTAGCCAGCGCTTTAGA  - CPSI_00R: TCTCTGAGCAAAAACACTGCGT  - CPSI_00R_C147G: TCTCTCAGCAAAAACACTGCGT  - CPSI_00P_MGB: ACAAAGACCTGGCGAGTA |
| Manufacturer of oligonucleotides | For the detection of *Ch. psittaci* by real-time PCR, the primers were part of the ready-to-use Kit RUO, manufactured by Nzytech, Portugal. |
| qPCR protocol | |

| Complete reaction conditions | According to manufacturer’s protocol:  Composition of the reaction mixture (15 µl final volume per reaction):  - 10 µl 2× Lyo NZYSupreme qPCR master mix;  - 1 µl C. psittaci primer/probe mix specific for CPSIT_0607;  - 1 µl Internal extraction control primer/probe mix;  - 3 µl RNase/DNase free water.  Reaction set-up:  - 15 µl of each reaction mix was pipetted into each well according to the real-time PCR experimental plate configuration;  - 5 µl of DNA template was pipetted into each well, according to the experimental plate set-up;  - negative control: 5 µl RNase/DNase free water instead of DNA template.  Thermal cycling conditions:  - polymerase activation (1 cycles): 2 min at 95 °C;  - denaturation \|annealing/extension (50 cycles): each of 5 s at 95 °C 30 s at 60 °C.  The amplification was carried out in Step One Real-Time PCR System (Applied Biosystems, Inc., Foster City (CA), USA). |
| --- | --- |
| Reaction volume and amount of DNA | Reaction volume: 20 µl  Amount of DNA: 5 µl |
| Kit identity and manufacturer | *Chlamydophila psittaci* Real-time PCR Kit, RUO (NZYtech, Portugal) cat. no.: MD03261 |
| Manufacturer of plates/tubes and catalog number | MicroAmp^TM^ Fast Optical 48-Well Reaction Plate (0,1 ml), PCR Compatibile DNA/RNA/RNase Free; Cat. No. 4375816,  MicroAmp^TM^ 48-Well Optical Adhesive Film, Compatibile DNA/RNA/RNase Free, Cat. No. 4275928 |
| Complete thermocycling parameters | - polymerase activation (1 cycles): 2 min at 95 °C;  - denaturation \| annealing/extension (50 cycles): each of 5 s at 95 °C 30 s at 60 °C. |
| Reaction set up | Manual |
| Manufacturer of qPCR instrument | StepOne Real-Time PCR System (Applied Biosystems, USA) |
| Data analysis | |
| qPCR analysis program (source, version) | StepOne^TM^ Software v2.0 (Applied Biosystems) |
| Software (source, version) | StepOne^TM^ Software v2.0 (Applied Biosystems) |
